# First person – Katrina Moore

**DOI:** 10.1242/bio.062294

**Published:** 2025-10-15

## Abstract

First Person is a series of interviews with the first authors of a selection of papers published in Biology Open, helping researchers promote themselves alongside their papers. Katrina Moore is first author on ‘ Beyond the burrow: Body condition and sex influence exploratory behavior in desert kangaroo rats (*Dipodomys deserti*)’, published in BiO. Katrina is a PhD candidate in the lab of Dr Monica Daley at UC Irvine, Irvine, USA, investigating how wildlife behaviour changes across different contexts and how these insights can guide effective conservation strategies.


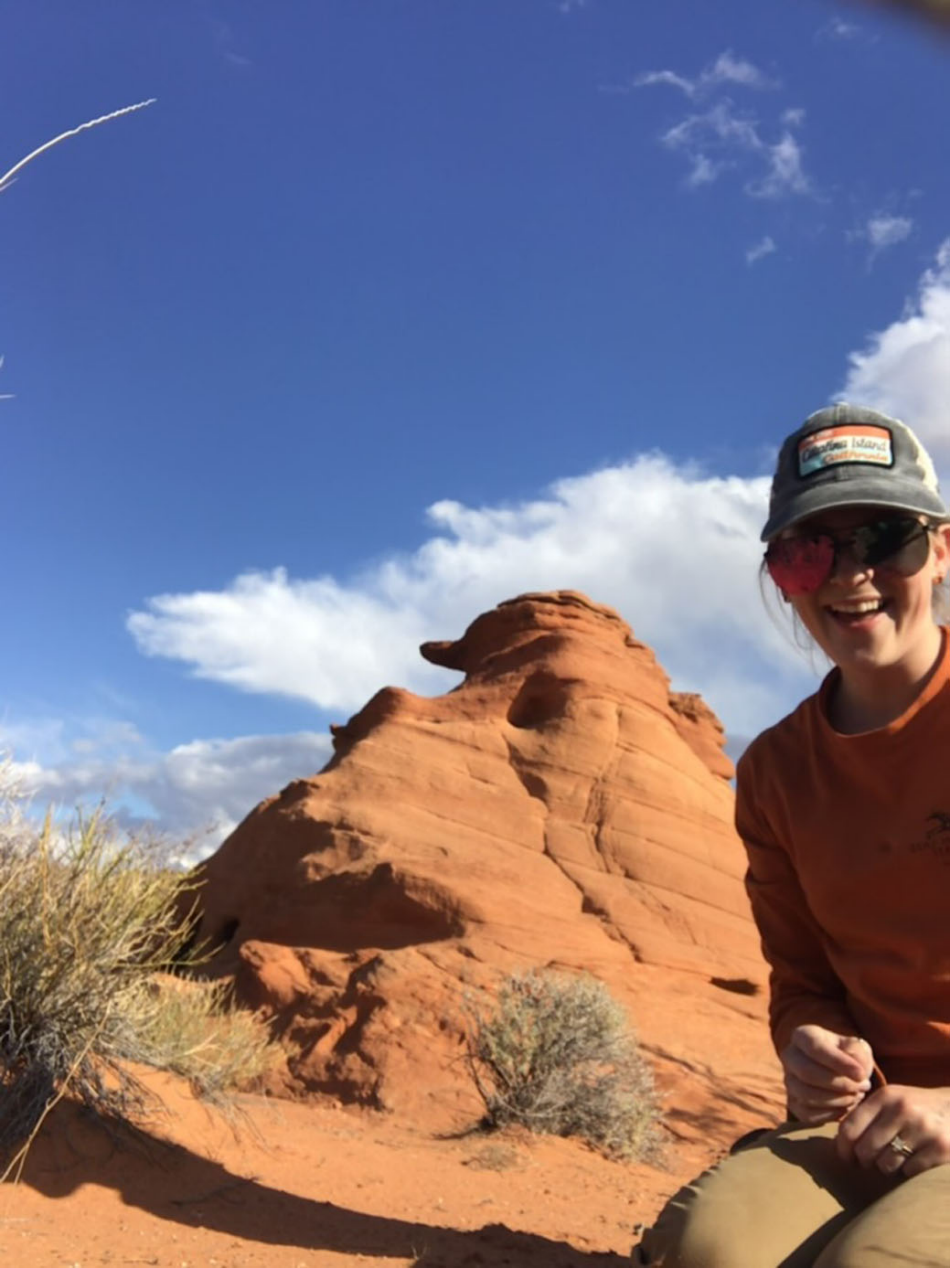


**Katrina Moore**

**Describe your scientific journey and your current research focus**

My path into science blends two passions: uncovering how animal anatomy and behaviour shape their lives and experiencing those dynamics firsthand in the field. As an undergraduate at Humboldt State University, I gained experience in research by studying cranial development in caecilians – eyeless, legless amphibians – under the guidance of Dr John Reiss. While that project cemented my passion for anatomy, I realised I wanted to take my curiosity outdoors and study animal behaviour in natural settings. That led me to a Master's degree at CSU Long Beach in Dr Ted Stankowich's mammal lab, where I explored how mammals navigate the joint pressures of predation and urbanisation. I was especially interested in whether two anatomically defended species – the striped skunk and the nine-banded armadillo – responded differently to risk given their unique anatomical defences. Now, as a fifth-year PhD candidate at UC Irvine in Dr Monica Daley's neuromechanics lab, my research focuses on how multiple types of environmental risk shape the behaviour and movement of wildlife. I'm approaching this question from multiple angles: first, by studying how desert kangaroo rats behave in open-field tests to uncover how individuals within a population vary in their responses to the same stimuli (this research is published in Biology Open alongside this interview), and next by using motion-triggered wildlife cameras to track the behaviour and movement of southern California's large carnivores – mountain lions, bobcats, coyotes, and gray foxes. With the camera trap data, I'm digging into how behaviour shifts across the landscape and teasing apart the risk factors that might drive those differences, from competition and prey density to human impact, terrain, and climate.

**Who or what inspired you to become a scientist?**

During my junior year of high school, I spent a year abroad in France with a host family. At the time, I thought I wanted to be a fashion journalist! But my host parents, Guy and Colette, quickly helped spark a different passion. Colette and I would take long walks through the forest near our small town, while Guy and I bonded over nature magazines and trips to visit his friends' farms. One day, Guy asked if I'd ever heard of Dian Fossey. I hadn't – but when he explained how she lived among gorillas and made groundbreaking discoveries about their behaviour, I was in awe. I hadn't realised a career like that even existed. From that moment, I devoured every book, documentary, and article I could find on field research. I didn't yet understand the long road ahead – graduate school, countless hours of work, and plenty of determination – but I knew I wanted a life where I could connect deeply with wildlife and contribute to protecting it.

**How would you explain the main finding of your paper?**

We discovered that desert kangaroo rats – small rodents with long hopping legs and tufted tails – don't all behave the same way, even when placed in the same outdoor enclosure. Some explored more, some less, and these differences seemed linked to things like whether the animal was male or female, how big it was, and even whether it was a new moon or a full moon.

**Figure:**
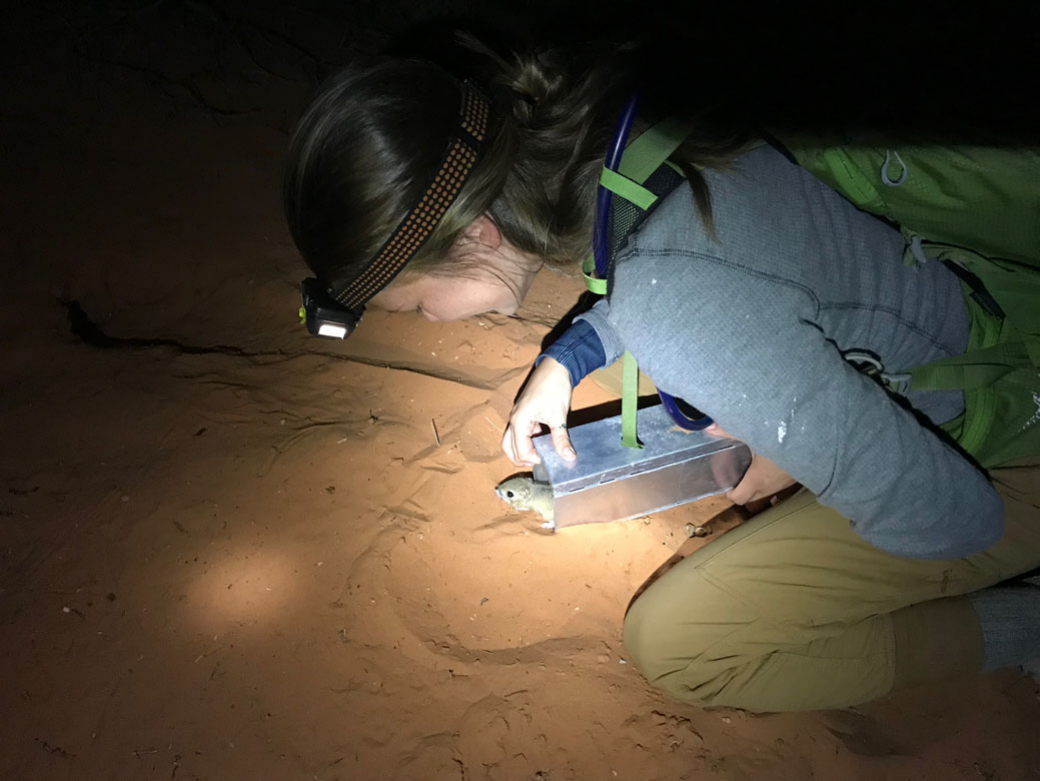
**Releasing a Merriam's kangaroo rat at our field site, one of the many curious visitors we caught during our study.** Since our project focused only on desert kangaroo rats, other species like this one were released immediately, often scampering off with their cheeks full of the sunflower seeds we used to bait the traps.

**What are the potential implications of this finding for your field of research?**

Anyone with a pet knows that no two animals act the same, and our study shows that's true for wild animals, too. In wildlife research, we often group species together and make broad conclusions, but important differences between individuals can get overlooked. Understanding these differences matters for conservation. For example, we found that male desert kangaroo rats tend to explore more and venture away from cover, while females stay closer to shrubs. While this species isn't currently threatened, if habitat restoration were ever needed, knowing these patterns could guide us to design habitats that support both sexes; for instance, making sure shrubs are placed in ways that allow females to feel safe while still finding food and potential mates.

**Which part of this research project was the most rewarding?**

My favourite part of this project was being out in the field. Our research site, with its red rocks and stunning sunsets, was breathtaking, and the work was as demanding as it was rewarding. We spent long days scouting trapping locations, set traps at dusk, and ran behaviour trials late into the night and early morning. Despite the long hours, the energy of fieldwork kept me going. Some of my best memories are the laughter and camaraderie I shared with my advisor and collaborator in this off-the-grid setting, something I know not all researchers get to experience with their mentors. And of course, I'll always be grateful to the desert kangaroo rats of Gold Butte for letting me glimpse their world and learn from them.

**What piece of advice would you give to the next generation of researchers?**

As tempting as it is, try not to compare yourself or your progress to others, everyone's path looks different. Love your work and work hard but remember you're also human. Take weekends to do something you enjoy and close your computer at the end of the day so you can recharge with something creative or restorative. You'll find you're not only more productive but also far more fulfilled when you make space to take care of yourself.
